# Ca^2+^-Activated Chloride Channels and Phospholipid Scramblases

**DOI:** 10.3390/ijms23042158

**Published:** 2022-02-15

**Authors:** Simone Pifferi, Anna Boccaccio

**Affiliations:** 1 Department of Experimental and Clinical Medicine, Università Politecnica delle Marche, 60126 Ancona, Italy; 2 Institute of Biophysics, Consiglio Nazionale delle Ricerche, 16149 Genova, Italy

The functional characterization of the TMEM16 protein family unexpectedly brought together two different research fields in membrane biology: anion channel and membrane lipid organization. Almost 40 years ago, Miledi described for the first time the presence of ion channels allowing the permeation of chloride ions activated by intracellular Ca^2+^ on the plasma membrane of *Xenopus laevis* oocytes [1]. In the same year, the investigation of platelets activation revealed the presence of Ca^2+^-dependent mechanisms mediating the exposure phosphatidylserine on the outer leaflet of the membrane dissipating the lipid asymmetry [2,3]. Later, the term “scramblase” was proposed for the proteins mediating this process. 

Further investigations shows that both Ca^2+^-activated Cl¯ channels (CaCCs) and scramblases are expressed in various tissues playing important physiological roles. In particular, CaCCs are involved in the secretion of different types of exocrine glands; regulate the contraction of vascular smooth muscle cells relevant to the modulation of blood pressure; control the chloride secretion in different epithelia functionally interacting with the cystic fibrosis transmembrane regulator (CFTR); and modulate the neuronal firing activity and the sensory transduction in olfactory systems [4,5]. Similarly, phospholipid scramblase has a pivotal role in the blood coagulation and in the removal of apoptotic cells [6].

In 2008 and 2010, the molecular identities of Ca^2+^-activated Cl¯ channels and phospholipid scramblases were discovered [7,8,9,10]. Different expression cloning approaches revealed that two members of the “transmembrane proteins with unknown function 16”, TMEM16, encode for CaCCs, and at least one member of the same family forms the phospholipid scramblase [7,8,9,10]. These seminal discoveries opened the possibility of investigation at the molecular level of CaCCs and phospholipid scramblases.

Today, we know that in mammals, the TMEM16 family, also known as the anoctamin family, is composed of 10 members with different functions and physiological roles (Figure 1) [11,12]. TMEM16A and B are classical CaCCs expressed mainly in epithelial and neuronal cell types, respectively [13]. TMEME16C, D, E, F, G, J and K are phospholipid scramblases [14,15,16]. Moreover, TMEM16D, E and F are also Ca^2+^-activated ion channels [17,18,19], and TMEM16J is an ion channel activated by the cAMP/PKA pathway [20]. Finally, TMEM16C modulates the activity of Na^+^-activated K^+^ channels [21]. Many of the TMEM16 proteins are involved in human diseases. In particular, mutations in *TMEM16C* cause craniocervical dystonia [22], *TMEM16E* cause gnatodiaphyseal dysplasia [23] and muscular dystrophy [24], mutations in *TMEM16F* are responsible for Scott syndrome [16], while a form of spinocerebellar ataxia is due to mutations in *TMEM16K* [25]. Moreover, many data show that TMEM16 is involved in cell proliferation and is overexpressed in several types of cancer [26]. TMEM16A can also contributes to pathogenesis of cystic fibrosis by a complex functional interplay with CFTR [27]. Finally, TMEM16E, G and J are also involved in several types of cancer [28,29,30,31].

In the Special Issue of *International Journal of Molecular Sciences* “Ca^2+^-Activated Chloride Channels and Phospholipid Scramblases”, we edited several papers bringing new light on the function of this interesting protein family.

Choi et al. reported that TMEM16A could be involved in psoriasis pathogenesis [32]. Psoriasis, affecting about 2% of the human population, is a multifactorial skin disease causing erythematous plaques, papules and pruritus [33]. Psoriatic skin shows both hyperplasia of the epidermis caused by over-proliferation of keratinocytes, and alteration in the proinflammatory response [33]. Choi et al. find that TMEM16A is overexpressed in psoriatic skin from human subjects. Pharmacological blockage and gene silencing of *TMEM16A* reduces the proliferation of the human keratocytes cell line HaCaT. Moreover, the inhibition TMEM16A decreased the psoriatic symptoms in a pharmacological-induced psoriasis mouse model. This effect could be partially due to a reduction of proinflammatory cytokines production and inhibition of AKT/ERK pathways [32]. These results confirm the relevant role on TMEM16A in cell proliferation and can be important to find new targets for psoriasis treatment.

Centerio et al. find that the CLCA1 protein controls the airway mucus production by modulating TMEM16A [34]. CLCA1 is a secreted protein that stabilizes and increases the membrane expression of TMEM16A in several tissues [35]. Therefore, the interplay between CLCA1 and TMEM16A represents a novel and interesting approach to modulate the CaCCs activity. Centerio et al. report that in mice, the application of CLCA in the airway does not increase the membrane expression of TMEM16A, however it provokes a significant increase in mucus production. Interestingly, mucus production mediated by CLCA1 application is further increased in the mouse model of asthma. Moreover, with an in vitro model of human airway epithelium, they show that mucus production induced by CLCA1 is dependent on TMEM16A expression without an increase of ion secretion. Finally, the proinflammatory cytokine IL-13 upregulates the expression of CLCA1, enhancing mucus production. These data provide a foundation for future work investigating the precise functional interaction between TMEM16A and CLCA1 in airway epithelia.

Seo et al. identified a new blocker for TMEM16A showing a proapoptic effect on lung cancer cells [36]. The pharmacology of the TMEM16 protein is still rudimental; very few specific blockers or agonists have been reported, and for most of them, the molecular mechanisms of blockage or activation are still unknown [37,38].

Using a high-throughput approach using the halide sensitive YFP, Seo et al. identified a new blocker of TMEM16A, diethelstilbestrol (DES). Unfortunately, DES also partially blocks TMEM16B. Considering that TMEM16A is overexpressed in some types of lung cancers [39], they screened several cell lines derived from human lung cancer for TMEM16A expression. PC9 cells show a high level of TMEM16A. DES significantly reduces the proliferation and migration of PC9 cells, whereas a smaller response is observed in H1975 cells lacking the TMEM16A expression. Interestingly, DES does not only block the current mediated by TMEM16A, but it also reduces the protein expression after chronic application for 72 h. Moreover, DES inhibits the EGFR and ERK pathway that are involved in TMEM16A-mediated cell proliferation [40,41]. Finally, they find that DES is able to induce apoptotic cell death in PC9 cells. These results show the possibility to pharmacologically control the TMEM16A expression and could be important to finding new targets for cancer treatment.

Ko and Suh investigated the role of membrane PI(4,5)P_2_ in controlling the TMEM16A gating [42]. Membrane lipids, and particularly the phosphoinositides, play a complex role in regulation of the TMEM16 proteins [43,44,45,46]. Here, Ko and Suh show that PI(4,5)P_2_ depletion inhibits TMEM16A depending on the splice variant. In particular, they find that only the isoform containing the exon c, coding a short stretch of four amino acids (EAVK), is inhibited by PI(4,5)P_2_ hydrolysis. This effect is specific for PI(4,5)P_2_, since it is not observed by reducing the concentration of PI(3,4,5)P_3_ and PI4P. Activation of PLC mediated cascade through the M1 muscarinic receptor induced the same effect of the PI(4,5)P_2_ depletion, indicating that this modulation could be physiologically relevant. This study again shows the intricated pathway controlling the gating of TMEM16A.

The complexity of the mechanisms controlling the opening of TMEM16A and other TMEM16 proteins is fully explored in the comprehensive review by Agostinelli and Tammaro [47]. They highlight how different stimuli, such as Ca^2+^, voltage, low extracellular pH, heat, membrane lipids, etc., regulate the gating of TMEM16 proteins [47]. They also review the recent structures of some TMEM16 proteins starting to build a model of TMEM16 gating.

The remaining two papers of this Special Issue deal with TMEM16F, a phospholipid scramblase that also mediates ion channel activity [48,49]. A still debated aspect of TMEM16F function as an ion channel is its ionic selectivity [50,51]. Indeed, even if all studies generally agree that TMEM16F is a poorly selective channel, some results obtained in whole-cell recordings from TMEM16F heterologously expressed in HEK-293 cells show a higher permeability to Cl¯ than Na^+^ [52,53,54]. In contrast, inside-out experiments indicate that TMEM16F is more permeable to cations than anions [19,55,56].

Stabilini et al. performed a detailed side-by-side comparison of electrophysiological properties of TMEM16F recorded in inside-out and whole-cell configuration [49]. They found that TMEM16F shows different behaviors depending on the recording method. In particular, in both conditions, TMEM16F is activated by µM of intracellular Ca^2+^, but in whole-cell configuration, TMEM16-meditated current develops with several seconds of delay that is not observed in inside-out. Moreover, they found that in whole-cell recordings, TMEM16F has a slight preference for anions; indeed, the permeability ration between Na^+^ and Cl (P_Na_/P_Cl_) is 0.4, whereas in inside-out, P_Na_/P_Cl_ is 3.7, indicating a higher Na^+^ permeability.

These results could, at least partially, be explained by the role of Ca^2+^ investigated by Nguyen et al. [48]. They found that Ca^2+^ and other divalents in the millimolar range can modulate TMEM16A and 16F-mediated current. This effect depends on the membrane concentration of PI(4,5)P_2_. Interestingly, in the Q559W mutant of TMEM16F, the intracellular application of millimoles of Ca^2+^ significantly reduced the permeability to Na^+^. Based on structural data, the author proposes that the gating of TMEM16A and 16F creates a groove in the protein big enough for the entry of PI(4,5)P_2_, where divalents can also enter, partially shielding the negative charges. The alteration of the local electrical field affects the ion selectivity. The bigger effects observed in TMEM16F could be due to its intrinsic lower selectivity with respect to TMEM16A. Further experiments will clarify this mechanism and the relevance for the function of other TMEM16 proteins.

All these new studies clearly show the complexity and versatility of the cellular processes mediated by Ca^2+^-activated chloride channels and phospholipid scramblases. We hope that in the near future we can gain a better understanding of TMEM16 physiology necessary to help treat the human diseases caused by TMEM16 mutation or mis-regulation.

## Figures and Tables

**Figure 1:**
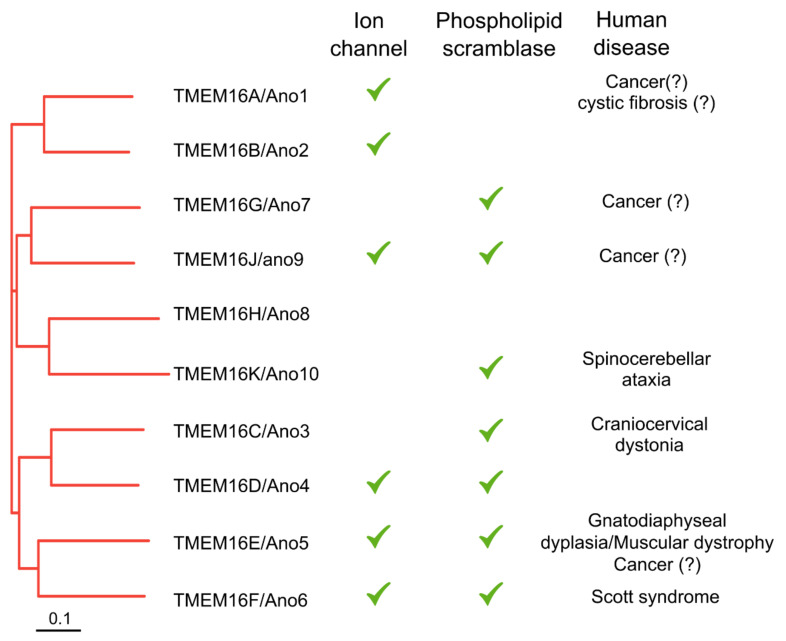
TMEM16 gene family. Phylogenetic tree showing the TMEM16 gene family in humans. Some members are ion channels, while some are phospholipid scramblases. At least TMEM16E and 16F are both scramblases and ion channels. Mutation or mis-regulation of some TMEM16 proteins cause human diseases. Scale bar, 0.1 nucleotide substitutions per site.

## Data Availability

Not applicable.
